# First Case Report of Sphingobium lactosutens as a Human Pathogen Causing Peritoneal Dialysis-Related Peritonitis

**DOI:** 10.7759/cureus.27293

**Published:** 2022-07-26

**Authors:** Sujith K Palleti, Santhoshi R Bavi, Margaret Fitzpatrick, Anuradha Wadhwa

**Affiliations:** 1 Nephrology, Loyola University Medical Center, Maywood, USA; 2 Internal Medicine, Ascension Saint Joseph Hospital, Chicago, USA; 3 Infectious Diseases, Edward Hines Jr. Veterans Administration Hospital, Hines, USA

**Keywords:** peritoneal dialysis-related peritonitis, resistance to colistin, antimicrobial susceptibility, biodegradative and biosynthetic capabilities, sphingobium paucimobilis, sphingobium olei, hexachlorocyclohexane dumpsite, sphingomonads, sphingobium lactosutens

## Abstract

Novel pathogens keep evolving from time to time. In this article, we describe a rare case of the bacterium* Sphingobium lactosutens* causing peritoneal dialysis-related peritonitis in a patient who presented to our dialysis clinic with typical features of abdominal pain and diffuse abdominal tenderness and was successfully treated with the intraperitoneal antibiotic therapy. There were only very few cases of infections caused by *Sphingobium* species before. Here, we discuss the infections caused by other *Sphingobium* species, probable sources of infection, clinical findings, and susceptibility patterns. We also aim to create awareness about this rare bacterial pathogen and emphasize the need for more research to successfully treat and prevent future infections.

## Introduction

*Sphingobium lactosutens* is a strictly aerobic, gram-negative, rod-shaped bacteria producing yellow- or off-white-pigmented colonies belonging to the genus “*Sphingomonas,*” which are sub-divided into several genera and are collectively referred to as “sphingomonads” [1]. *S. lactosutens* was first isolated from hexachlorocyclohexane dumpsite in 2009 [2] and has never been reported to be isolated from human beings. Peritonitis is a common complication of peritoneal dialysis (PD) and is associated with significant morbidity, catheter loss, and even death if not treated properly. PD-related peritonitis is usually due to contamination by skin bacterial pathogens during exchanges or an exit-site or tunnel infection. To date, very few cases of infections caused by *Sphingobium* in humans have been reported, with *S. paucimobilis*, *S. yanoikuyae*, *S. olei*, and *S. paucimobilis* being the most commonly reported [3]. In addition, little is known about their sensitivity patterns.

## Case presentation

A 52-year-old man with end-stage renal disease secondary to long-standing type 2 diabetes and hypertension on PD for four years presented to our dialysis center with complaints of abdominal pain for four days in addition to fatigue and early satiety. On physical examination, all vitals were stable. There was diffuse tenderness of the abdomen on palpation, with no rebound or guarding. The peritoneal catheter exit site and tunnel tract were clean without any signs of infection. PD fluid was sent for cell count, gram stain, and culture, and the patient was started on broad-spectrum intraperitoneal (IP) antibiotic coverage with vancomycin and ceftazidime for possible peritonitis. PD fluid resulted in a total WBC count of 5194 with 95% polymorphonuclear neutrophils (PMNs), and culture results showed gram-negative bacilli. Vancomycin was stopped and IP ceftazidime was continued for two weeks.

**Figure 1:**
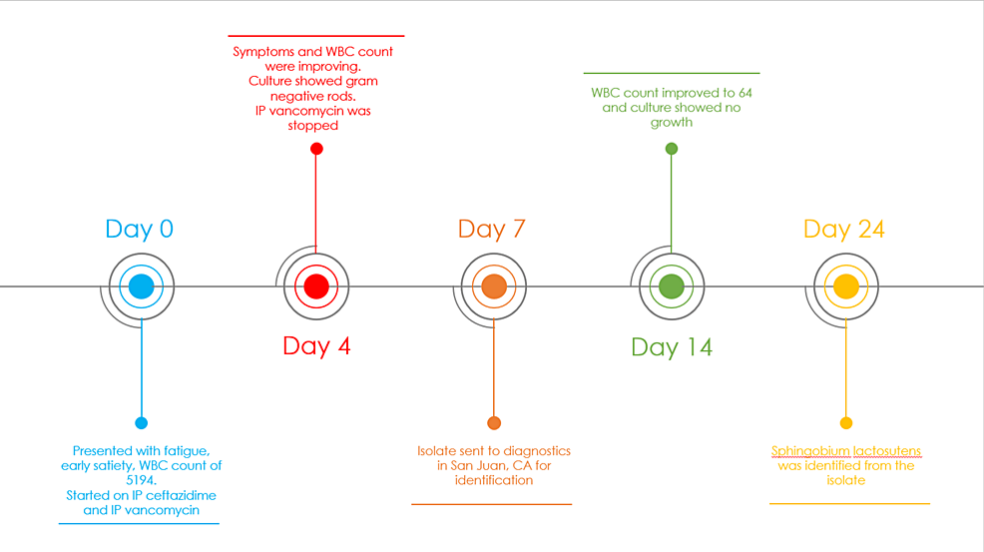
Timeline from presentation to identification of the bacterium. IP: intraperitoneal.

Abdominal pain improved and a repeat peritoneal fluid culture showed no growth after three days of antibiotics, and the PD fluid WBC count progressively declined over two weeks. The isolate was sent out to Quest Diagnostics in San Juan, California on day seven, which was later speciated as *S. lactosutens* on day 24 (Figure 1).

## Discussion

The most common symptom of peritonitis among PD patients is abdominal pain, seen in almost 88% of the population [4]. Other common symptoms include fever, nausea, vomiting, diarrhea, and hypotension, which may or may not be present in all cases. In our patient, abdominal pain was the predominant finding with diffuse abdominal tenderness. Clinical findings of peritonitis were confirmed with laboratory results showing the increase in the peritoneal fluid leukocyte count and positive culture report, which showed gram-negative bacilli and later isolated as *S. lactosutens. *The organism was identified by bacterial 16S ribosomal RNA sequencing using a laboratory-developed assay, which has been validated under the Clinical Laboratory Improvement Amendments (CLIA) regulations.

The patient was initially started on broad-spectrum IP antibiotic coverage with vancomycin and ceftazidime, which was narrowed down later to IP ceftazidime following culture results. There was a gradual improvement of the clinical condition and resolution of infection, which was also evident in laboratory findings with reduced peritoneal fluid WBC count and negative culture results later.

Historically, sphingomonads have been used for a wide range of biotechnological applications due to their biodegradative and biosynthetic capabilities [2]. They can be found in medical devices, contaminated fluid, and respirators in the hospital [5]. According to Lin et al., hospital-acquired bacteremia accounted for 69.0% of infections [3]. There was a single case report of peritonitis caused by *S. olei*, which was resistant to IP ceftazidime and tobramycin, leading to the eventual removal of the PD catheter [6]. Intrinsic resistance to colistin was observed in 92% of the genera [7]. The highest antibiotic resistance was observed in members belonging to genera *Sphingomonas* and *Sphingobium* for beta-lactams, ciprofloxacin, and cotrimoxazole. The most effective antibiotics against *S. paucimobilis *were fluoroquinolones, carbapenems, and trimethoprim/sulfamethoxazole [8].

## Conclusions

As per our knowledge, this is the first case report of *S. lactosutens* as a human pathogen. While our patient recovered from the infection, there are still limited data available regarding antimicrobial susceptibility, and it can be challenging for clinicians to treat this rare bacterium. We highlight the need for increasing awareness of infections associated with this rare organism, especially in patients on PD who are at high risk for severe infections.
